# Immune Escape Adaptive Mutations in Hemagglutinin Are Responsible for the Antigenic Drift of Eurasian Avian-Like H1N1 Swine Influenza Viruses

**DOI:** 10.1128/jvi.00971-22

**Published:** 2022-08-02

**Authors:** Chengzhi Xu, Naixin Zhang, Yuying Yang, Wenhua Liang, Yaping Zhang, Jingfei Wang, Yasuo Suzuki, Yunpu Wu, Yan Chen, Huanliang Yang, Chuanling Qiao, Hualan Chen

**Affiliations:** a State Key Laboratory of Veterinary Biotechnology, Harbin Veterinary Research Institute, Chinese Academy of Agricultural Sciences, Harbin, China; b Department of Medical Biochemistry, University of Shizuoka School of Pharmaceutical Sciences, Shizuoka, Japan; St. Jude Children’s Research Hospital

**Keywords:** antigenicity, EA H1N1, escape mutant, HA protein, swine influenza virus

## Abstract

The continuous antigenic variation of influenza A viruses remains a major hurdle for vaccine selection; however, the molecular determinants and mechanisms of antigenic change remain largely unknown. In this study, two escape mutants were generated by serial passages of the Eurasian avian-like H1N1 swine influenza virus (EA H1N1 SIV) A/swine/Henan/11/2005 (HeN11) in the presence of two neutralizing monoclonal antibodies (mAbs) against the hemagglutinin (HA) protein, which were designated HeN11-2B6-P5 and HeN11-4C7-P8, respectively. The HeN11-2B6-P5 mutant simultaneously harbored the N190D and I230M substitutions in HA, whereas HeN11-4C7-P8 harbored the M269R substitution in HA (H3 numbering). The effects of each of these substitutions on viral antigenicity were determined by measuring the neutralization and hemagglutination inhibition (HI) titers with mAbs and polyclonal sera raised against the representative viruses. The results indicate that residues 190 and 269 are key determinants of viral antigenic variation. In particular, the N190D mutation had the greatest antigenic impact, as determined by the HI assay. Further studies showed that both HeN11-2B6-P5 and HeN11-4C7-P8 maintained the receptor-binding specificity of the parent virus, although the single mutation N190D decreased the binding affinity for the human-type receptor. The replicative ability *in vitro* of HeN11-2B6-P5 was increased, whereas that of HeN11-4C7-P8 was decreased. These findings extend our understanding of the antigenic evolution of influenza viruses under immune pressure and provide insights into the functional effects of amino acid substitutions near the receptor-binding site and the interplay among receptor binding, viral replication, and antigenic drift.

**IMPORTANCE** The antigenic changes that occur continually in the evolution of influenza A viruses remain a great challenge for the effective control of disease outbreaks. Here, we identified three amino acid substitutions (at positions 190, 230, and 269) in the HA of EA H1N1 SIVs that determine viral antigenicity and result in escape from neutralizing monoclonal antibodies. All three of these substitutions have emerged in nature. Of note, residues 190 and 230 have synergistic effects on receptor binding and antigenicity. Our findings provide a better understanding of the functional effects of amino acid substitutions in HA and their consequences for the antigenic drift of influenza viruses.

## INTRODUCTION

Swine influenza is an acute respiratory disease caused by swine influenza virus (SIV). Pigs have been proposed to be a “mixing vessel” of influenza viruses due to the presence of human- and avian-like sialic acid receptors on their cells, which play important roles in the transmission of viruses and the generation of new strains (1, 2). Multiple lineages of H1N1, H1N2, and H3N2 SIVs cocirculate in swine populations (3, 4). Avian-like H1N1 SIV was first isolated from pigs in 1979 in Belgium (5) and has since spread to many European and Asian countries (6–8); for this reason, it is referred to as Eurasian avian-like H1N1 SIV (EA H1N1 SIV). After the reintroduction of the human pandemic 2009/H1N1 virus to pigs, multiple genotypes of reassortant H1N1 and H1N2 SIVs bearing the hemagglutinin (HA) gene of EA H1N1 SIV have replaced the pure EA H1N1 SIV and are currently prevalent in swine populations in China (9–13). Sporadic infections caused by EA H1N1 or EA H1N1-like viruses and antigenic variation within these viruses pose new potential threats to public health and also present new challenges to the pig industry (10, 12, 14–19).

HA is the key surface glycoprotein of influenza viruses that mediates infection. Antibodies against HA generally neutralize viral infectivity, presumably by interfering with either virus attachment to sialic acid receptors on the host cell surface or the subsequent process of fusion between the virus and endosomal membrane (20–22). Antigenic shift, caused by gene reassortment involving HAs of different subtypes, and antigenic drift, caused mainly by gene mutation in HA, have been widely documented (23–25). Antigenic drift, which plays a pivotal role in the evolution and persistence of influenza viruses in various animal and human populations, leads to the gradual accumulation of point mutations in epitopes or antibody-binding regions. A drifted strain usually carries mutations in several major epitopes of the viral HA surface protein. Several epitopes located around the receptor-binding site of the HA head region (including Sa, Sb, Ca1, Ca2, and Cb) have been proposed as antigenic sites of H1N1 HA based on variants selected by monoclonal antibodies (mAbs) (26, 27).

The targets of antiviral immunity are located mainly on the HA glycoprotein; therefore, it is important to reveal the antigenically relevant parts of the HA molecule that are prone to amino acid substitutions that lead to the acquisition of resistance to neutralizing antibodies. We previously characterized two murine mAbs against the HA of an EA H1N1 SIV (A/swine/Henan/11/2005 [HeN11]) with neutralizing and hemagglutination inhibition (HI) activities that provide complete protection when administered passively to mice prior to challenge with the homologous virus (28). In the present study, escape mutants of HeN11 were generated via virus propagation in the presence of mAbs to identify the key amino acid residues that determine the antigenicity of EA H1N1 SIV. In addition, the impacts of escape mutations on cross-reactive mAb neutralization, antigenicity, receptor-binding specificity or affinity, and viral replication *in vitro* were also evaluated.

## RESULTS

### Generation of escape mutants.

To identify the key residues of the epitopes recognized by the two neutralizing mAbs, escape mutants of HeN11 were selected by coculturing the virus with the mAbs. Variants were established when similar levels of growth were obtained in the presence and absence of the mAbs; that is, the virus was no longer neutralized. One variant was generated by the coculturing of HeN11 with mAb 2B6 for five passages and was designated HeN11-2B6-P5. The other variant, designated HeN11-4C7-P8, was generated by the coculturing of HeN11 with mAb 4C7 for eight passages. The complete genomes of the escape mutants were sequenced, and amino acid point mutations were identified by sequence comparisons to the wild-type HeN11 virus. The HeN11-2B6-P5 mutant harbored the N190D and I230M substitutions in HA (H3 numbering), and the HeN11-4C7-P8 harbored the M269R mutation in HA (H3 numbering). Selection for an additional three passages identified no further mutations. No passage-related mutation was detected in the viruses incubated in the absence of the mAbs.

### Location of the mutated amino acid residues in the HA structure.

**FIG 1:**
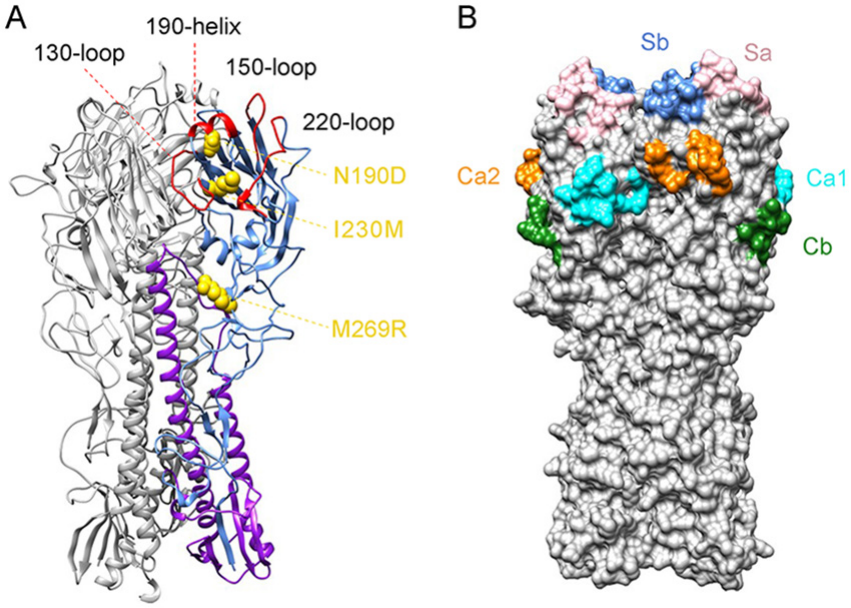
Homology model of HA and location of the mutated amino acid residues in the HA structure. (A) One promoter of the trimeric HA, with HA1 in cornflower blue, HA2 in purple, and the three mutated residues shown as gold spheres. The other two HAs are in gray. The 190 helix, 130 loop, 150 loop, and 220 loop of the receptor-binding site are labeled and shown in red. (B) The surface of the HA trimer. Highlighted sites are Sa (pink), Sb (cornflower blue), Ca1 (cyan), Ca2 (orange), and Cb (forest green).

To locate the mutated amino acid residues in the structure of the HA protein, homology models of the HA protein of HeN11 with the three substitutions (N190D, I230M, and M269R) were constructed by using the SWISS-MODEL online server (https://swissmodel.expasy.org). Ramachandran plots revealed that 97.12% of the residues were located in favored regions, indicating that the model was of high quality. The residue at position 190 is located in helix 190 of the receptor-binding site, whereas the residue at position 230 is located at the bottom of the receptor-binding site. Both residues belong to antigenic site Sb of H1 HAs (Fig. 1). The residue at position 269 is located in the head domain of HA and not in any antigenic regions identified previously in the H1 HAs but instead is located in the region corresponding to antigenic site C of H3 HAs (Fig. 1).

### N190D and M269R are key mediators of antibody binding of the escape mutants.

The HeN11-2B6-P5 mutant carries a double mutation of N190D and I230M in HA. To explore whether both residues acted as functional mediators of antibody binding, we used reverse genetics to rescue the recombinant HeN11 (rHeN11) virus and the recombinant mutants rHeN11/N190D and rHeN11/I230M by introducing the N190D and I230M substitutions, respectively, into the HA of HeN11. We then tested their reactivities with mAb 2B6 in a neutralization assay. As shown in Table 1, the neutralization titer of the mAb against the rHeN11/N190D mutant was 40, which was 16-fold lower than that against the rHeN11 virus. However, there was only a 2-fold reduction in the neutralization titer of the mAb against the rHeN11/I230M mutant, in contrast to the rHeN11 virus (Table 1). In the HeN11-4C7-P8 mutant, the single mutation M269R in HA abolished the ability of mAb 4C7 to bind the virus. These results demonstrate that N190D and M269R are key mediators of the antibody-binding abilities of the escape mutants and that I230M is probably a concomitant compensatory mutation acquired during the generation of the escape mutants.

**TABLE 1:** Effects of the amino acid substitution(s) on antigenic variation tested by using a mAb and polyclonal sera^*a*^

Virus	Neutralization antibody titer of mAb 2B6^*c*^	HI antibody titer of antisera induced by different viruses^*b*^							
		rHeN11		rHeN11/N190D		rHeN11/PR8		rHeN11/N190D/PR8	
		Chicken sera	Pig sera	Chicken sera	Pig sera	Chicken sera	Pig sera	Chicken sera	Pig sera
rHeN11	**640**	**1,024**	**2,560**	256	160	512	640	256	160
HeN11-2B6-P5	<10	128	160	1,024	1,280	64	160	512	640
HeN11-4C7-P8	NT	512	1,280	128	320	512	1,280	128	160
rHeN11/N190D	40	64	160	**1,024**	**1,280**	64	320	512	640
rHeN11/I230M	320	512	1,280	256	160	256	1,280	256	320
rHeN11/PR8	NT	512	1,280	256	320	**1,024**	**1,280**	128	320
rHeN11/N190D/PR8	NT	64	320	512	1,280	64	160	**1,024**	**1,280**

a The antisera were generated by vaccinating specific-pathogen-free chickens or pigs with the indicated oil-emulsified inactivated viruses.

b The homologous titers are shown in boldface type.

c NT, not tested.

### Validation of the antigenic effects of the amino acid substitutions.

First, we tested whether the antibodies induced by the rHeN11 virus still recognized the two escape mutants HeN11-2B6-P5 and HeN11-4C7-P8. Postvaccination sera were generated by inoculating chickens and pigs with the rHeN11 vaccine and tested in an HI assay using the rHeN11 virus and the two escape mutants HeN11-2B6-P5 and HeN11-4C7-P8 as antigens. As shown in Table 1, the HI antibody titers of the HeN11-2B6-P5 mutant with the rHeN11-vaccinated sera from chickens and pigs were 8- and 16-fold lower than the homologous titers. However, the HeN11-4C7-P8 mutant reacted well with the two sera, and the titers were only 2-fold lower than the homologous ones (Table 1). To further determine whether N190D or I230M in the HA of the HeN11-2B6-P5 mutant led to the evasion of recognition by serum antibodies, we performed the HI assay using the rHeN11/N190D and rHeN11/I230M viruses as antigens. The HI titers of the rHeN11/N190D virus with the chicken and pig sera were 16-fold lower than the homologous titers. However, compared to those of the rHeN11 virus, there was a 2-fold reduction in the HI antibody titer of the rHeN11/I230M virus with the two sera (Table 1).

Next, we prepared sera against the rHeN11/N190D virus and performed HI cross-reactions with the test viruses. As shown in Table 1, each serum sample reacted well with the HeN11-2B6-P5 mutant, but the antibody titers against the rHeN11, HeN11-4C7-P8, and rHeN11/I230M viruses were reduced by 4- to 8-fold. Taken together, these results demonstrate that the double mutation of N190D and I230M in HA decreases the reactivity of the HeN11-2C6-P5 mutant with antibodies induced by the rHeN11 virus, whereas the single mutation of M269R is insufficient to reduce the interaction of the HeN11-4C7-P8 mutant with sera against the rHeN11 virus, and the N190D substitution in HA is the key factor influencing viral antigenicity.

To eliminate the impact of other viral proteins on the HI assay, we used the A/Puerto Rico/8/1934 (H1N1) (PR8) virus as a backbone and rescued HeN11/PR8 (1+7) recombinants bearing either the wild-type or mutated HeN11HA gene harboring the single mutation N190D, and the other seven genes of PR8 virus, as described previously (29), which we named rHeN11/PR8 and rHeN11/N190D/PR8, respectively. We then performed HI tests using polyclonal antisera against these two recombinant viruses (Table 1). Compared to those of the rHeN11/PR8 virus, there were 16- and 8-fold reductions in the HI titers of the rHeN11/N190D/PR8 virus with the rHeN11/PR8-vaccinated chicken and pig sera. Conversely, the HI titers of the rHeN11/PR8 virus with the two rHeN11/N190D/PR8-vaccinated sera were 8- and 4-fold lower than the homologous titers (Table 1). These data confirm that the N190D mutation in HA has a high impact on the antigenic variation of the EA H1N1 viruses.

### Effects of amino acid changes in the escape mutants on viral receptor-binding preference.

**FIG 2:**
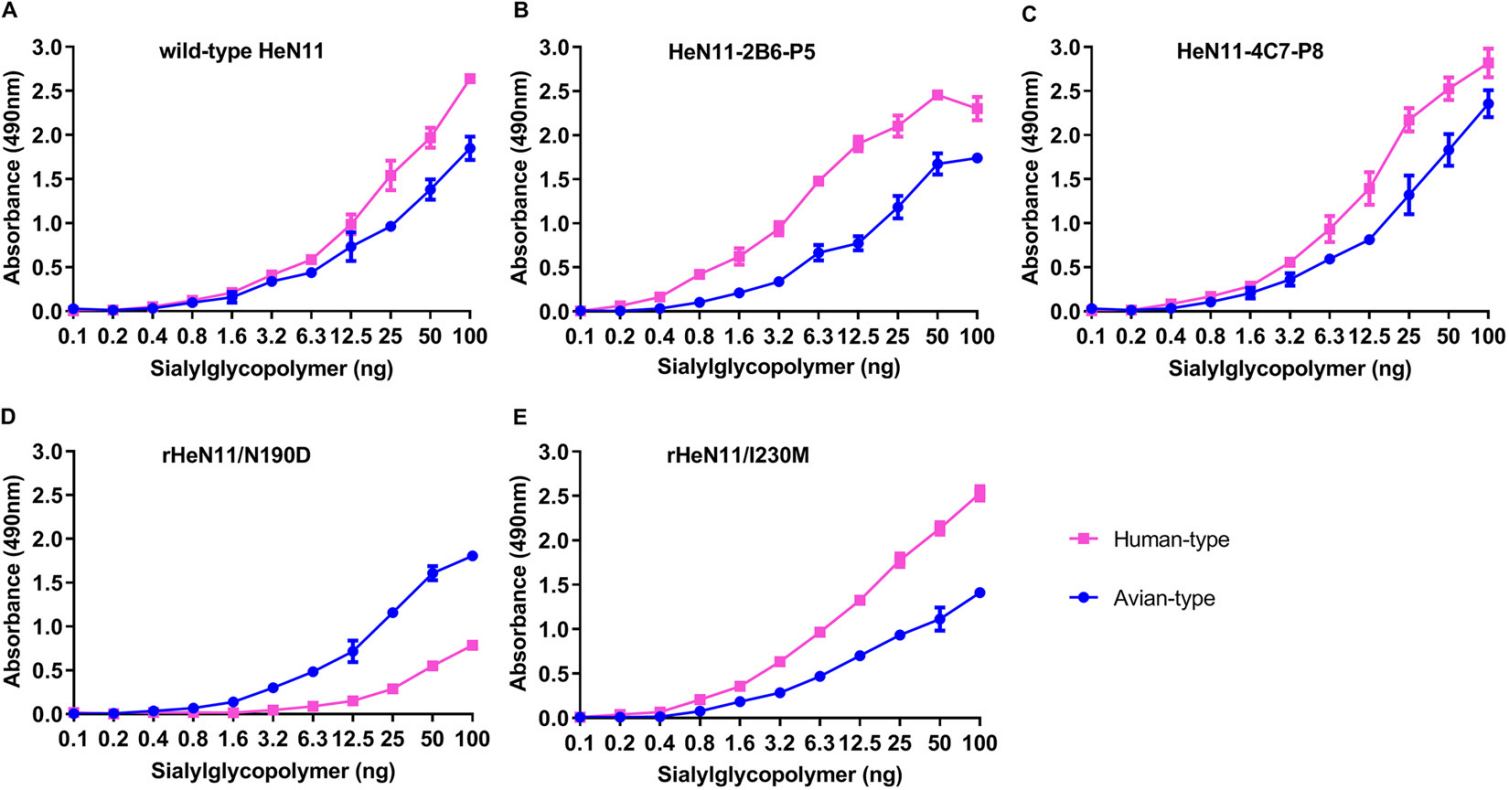
Receptor-binding properties of the indicated viruses. The receptor-binding specificity was tested using two different glycopolymers: an α-2,3-sialylglycopolymer (Neu5Acα2-3Galβ1-4GlcNAcβ1-pAP [*para*-aminophenyl]-α-PGA [α-polyglutamic acid]) and an α-2,6-sialylglycopolymer (Neu5Acα2-6Galβ1-4GlcNAcβ1-pAP-α-PGA). Chicken antisera against the indicated viruses were used as the primary antibodies, and a horseradish peroxidase-conjugated goat anti-chicken antibody was used as the secondary antibody. The absorbance was measured at a wavelength of 490 nm. The data are presented as the means ± SD from three replicates. (A) Wild-type HeN11 virus. (B) Escape mutant virus HeN11-2B6-P5. (C) Escape mutant virus HeN11-4C7-P8. (D) Rescued virus rHeN11/N190D. (E) Rescued virus rHeN11/I230M.

To determine if the amino acid substitutions of the escape mutants altered the viral receptor-binding preference, a solid-phase binding assay was performed to test the receptor-binding properties of the wild-type HeN11 virus and the two escape mutants. The escape mutants HeN11-2B6-P5 and HeN11-4C7-P8 maintained the same receptor-binding properties as those of the wild-type HeN11 virus, binding to both the avian- and human-type receptors with high affinity (Fig. 2A to C). We then tested the receptor-binding preferences of the rHeN11/N190D and rHeN11/I230M mutants. Compared with the wild-type HeN11 virus, the rHeN11/N190D virus showed a decreased binding affinity for the human-type receptor (Fig. 2D), whereas the I230M mutation had no impact on the receptor-binding preference (Fig. 2E). These results indicate that the N190D mutation in HA decreases the affinity of EA H1N1 SIV for the human-type receptor, and while the I230M substitution *per se* has no effect on receptor-binding properties, in the presence of the N190D mutation, it might synergistically restore the binding affinity of the HeN11-2B6-P5 mutant for the human-type receptor.

### Replicative abilities of the escape mutants.

**FIG 3:**
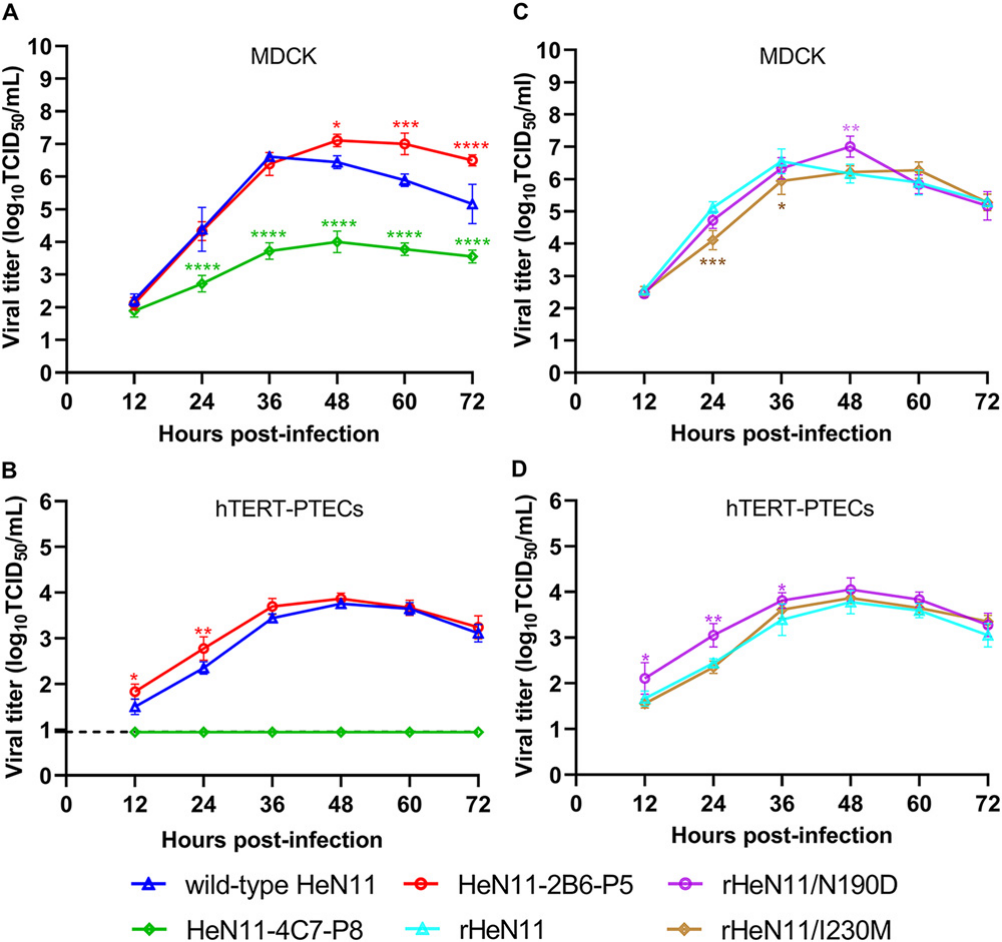
Replication kinetics of wild-type HeN11 and mutant viruses *in vitro*. MDCK cells and hTERT-PTECs were infected at multiplicities of infection of 0.001 and 0.01. Supernatants were collected at 12, 24, 36, 48, 60, and 72 hpi, and the virus titers were determined by a TCID_50_ assay in MDCK cells. (A and B) The wild-type HeN11 virus and two escape mutants in MDCK cells and hTERT-PTECs. (C and D) The three rescued viruses in MDCK cells and hTERT-PTECs. Each data point on the curve indicates the mean ± SD from three independent experiments. *, *P < *0.05; **, *P < *0.01; ***, *P < *0.001; ****, *P < *0.0001. The dashed lines indicate the lower limits of detection.

To determine whether both escape mutants maintained a level of replicative ability similar to that of the parent HeN11 virus, we examined virus growth kinetics in Madin-Darby canine kidney (MDCK) cells and human telomerase reverse transcriptase-immortalized porcine tracheal epithelial cells (hTERT-PTECs), which were prepared by transfecting primary PTECs with hTERT (30). Compared with that of the parent HeN11 virus, the replication of the HeN11-2B6-P5 mutant was significantly increased in MDCK cells in the late stage of infection (i.e., 48 to 72 h postinfection [hpi]) (Fig. 3A). In contrast, the replication of the HeN11-4C7-P8 mutant was significantly decreased at all time points tested except 12 hpi (Fig. 3A). Interestingly, the HeN11-2B6-P5 mutant displayed significantly increased replication compared with that of the parent HeN11 virus in hTERT-PTECs at 12 and 24 hpi, whereas the replication of the HeN11-4C7-P8 mutant was completely impaired at all time points tested (Fig. 3B).

To determine the effect of each mutated residue on the replicative ability of the escape mutant HeN11-2B6-P5, the replicative capacities of the parent virus and the rescued rHeN11/N190D and rHeN11/I230M viruses were also compared in MDCK cells and hTERT-PTECs. In MDCK cells, both of the rHeN11/N190D and rHeN11/I230M mutants maintained replication characteristics similar to those of the rHeN11 virus at each time point, except at 48 hpi for rHeN11/N190D and 24 and 36 hpi for rHeN11/I230M (Fig. 3C). In hTERT-PTECs, the rHeN11/N190D mutant displayed significantly increased replication in the early stage of infection (i.e., 12 to 36 hpi) compared with the rHeN11 virus. In contrast, no significant difference in replication was observed between the rHeN11/I230M and rHeN11 viruses (Fig. 3D).

### Escape mutants have emerged in nature.

**FIG 4:**
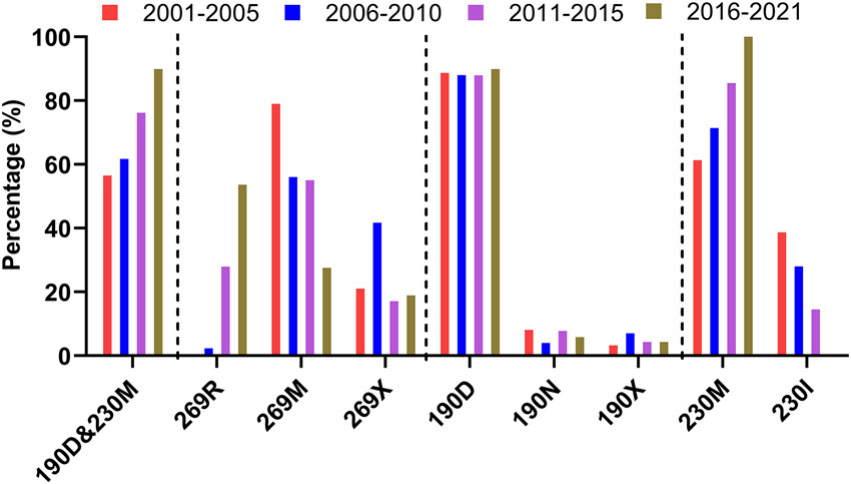
Polymorphism analysis of the mutated residues in the escape mutants of the HeN11 virus. A total of 768 H1 HA sequences of influenza viruses, including branches of 1C1, 1C1-2-like, 1C2, 1C2-like, 1C2.1, 1C2.2, 1C2.2-3-like, and 1C2.3, isolated from 2001 to 2021 were downloaded from the Influenza Research Database (https://www.fludb.org/brc/home.spg?decorator=influenza [up to 29 November 2021]). These sequences were then subjected to multiple-sequence alignments of the corresponding regions of the HA proteins by using the MAFFT multiple-sequence alignment program (https://mafft.cbrc.jp/alignment/software/). X indicates amino acid residues at the indicated positions other than those found in the HeN11 parent virus and escape mutants.

To investigate the polymorphism of each mutated residue detected in the escape mutants, all EA H1 HA sequences of influenza viruses, irrespective of host origin, including branches of 1C1, 1C1-2-like, 1C2, 1C2-like, 1C2.1, 1C2.2, 1C2.2-3-like, and 1C2.3, from 2001 to 2021 at 5-year intervals were downloaded from the Influenza Research Database (https://www.fludb.org/brc/home.spg?decorator=influenza [up to 29 November 2021]). Multiple-sequence alignments of the amino acid sequences of the corresponding regions of the HA proteins were conducted using the MAFFT multiple-sequence alignment program (https://mafft.cbrc.jp/alignment/software/). The two mAb escape mutations, including the double-amino-acid substitution of N190D and I230M and the single mutation of M269R, were found in naturally isolated influenza viruses bearing EA lineage H1 HA (Fig. 4). The percent representation of the HA double mutation of 190D and 230M had gradually increased from 56.5% in 2001 to 89.9% in 2021. The amino acid residue at position 269 of the analyzed sequences varied, with six different amino acid residues besides M and R (designated X in Fig. 4). A virus carrying the HA mutation 269R was first isolated in China in 2010. The prevalence of 269R increased from 2.3% to 53.6% and is now the most common mutation (Fig. 4). Further analysis of the amino acid frequencies at positions 190 and 230 revealed that the most common mutations at position 190 of HA were D, N, V, and S, with 190D accounting for about 88.6%. At position 230, there were two amino acids, 230I and 230M. The percentage of 230M gradually increased from 61.3% in 2001 to 100% in 2021, having completely replaced 230I in 2016 (Fig. 4).

## DISCUSSION

Influenza viruses continually escape antibody-mediated neutralization by changing the amino acid residues in their HA head domain, which induces antigenic drift. Various methods to quantify antigenic drift, as well as site-directed mutagenesis, have revealed that substantial antigenic drift can be caused by one or a few amino acid substitutions adjacent to the functional sites in the head, but not the stalk, of HA (31–33). In the present study, two escape mutants, HeN11-2B6-P5 and HeN11-4B7-P8, were generated by serial passages of the HeN11 virus in MDCK cells in the presence of neutralizing mAbs. The HeN11-2B6-P5 mutant simultaneously carried two mutations, at positions 190 and 230, and the HeN11-4B7-P8 mutant harbored a single mutation, at position 269. Mapping of the HA epitopes revealed that all three mutated residues were located in the head domain of HA. In general, a variant is considered antigenically different when the neutralization titer is reduced by more than 8-fold relative to that of a reference strain (34). In our study, the HeN11-2B6-P5 mutant escaped from mAb 2B6 and the HeN11-4B7-P8 mutant escaped from mAb 4C7 when screened by using a neutralization assay. To further screen the antigenicity-associated amino acid sites, the antigenicities of the two escape mutants were analyzed by neutralization and HI assays using the mAbs 2B6 and 4C7 as well as antisera against the corresponding viruses. The results suggest that the double mutation of N190D and I230M in HA or the single mutation of M269R in HA is sufficient to induce the antigenic drift of EA H1N1 SIV, which is consistent with previous findings that major antigenic changes in seasonal human influenza viruses are due to amino acid substitutions immediately adjacent to the receptor-binding site (32).

Antigenic changes that are sometimes due to a lower affinity for sialic acid and substitutions outside the antigenic motif may be compensatory or simply hitchhiker mutations (35–37). Various amino acid substitutions in the receptor-binding-site regions have been reported to affect the affinity of HA for sialic acid receptors (38–40). In particular, residue 190 plays a prominent role in the receptor-binding preferences of the pandemic 1918/H1N1, avian H1N1, and pandemic 2009/H1N1 viruses (41–44). In the present study, we found that the amino acid substitution N190D reduced the affinity for the human-type sialic acid receptor, which indicates that residue 190 also affects the virus receptor-binding preference of EA H1N1 SIV. In our study, we also found that the rHeN11/I230M mutant harboring a single mutation at position 230 in HA possessed the same reactivity with the mAbs and polyclonal antisera used in the neutralization and HI assays, which demonstrates that a compensatory mutation was induced during the generation of the mAb escape mutant.

The antigenic drift of influenza viruses usually comes with a fitness cost or produces some concomitant effects (45). Therefore, it is important to evaluate the fitness of escape mutants to understand the impact of each mutation in the globular head region of HA. Accordingly, we performed viral growth curve experiments using MDCK cells and hTERT-PTECs and found that the replicative abilities *in vitro* of the HeN11-2B6-P5 mutant were enhanced at different stages of infection in these two cell types. In contrast, the replicative abilities of the HeN11-4C7-P8 mutant were reduced compared with those of the parent virus. Thus, these two escape mutants showed different fitness changes when their replication kinetics were evaluated, and the underlying mechanism requires further study.

The mechanisms of immune escape of influenza viruses are sometimes ascribed to amino acid substitutions that alter the biophysical properties of an epitope and have the potential to cause antigenic change by directly affecting antibody binding. Amino acid substitutions that lead to an additional N-linked glycosylation site in HA have been shown to affect the antigenicity of influenza viruses by masking the antigenic epitopes in the globular region (29, 46, 47). The single-amino-acid substitution G158E in the HA of EA H1N1 SIV was reported previously to alter viral antigenicity, probably due to steric effects (48). In addition, amino acid substitutions that modulate receptor-binding avidity can also contribute to apparent antigenic changes as detected by the HI assay (37, 48). Modulation of avidity has been proposed as a true form of immune escape rather than an artifact of the HI assay (35, 36, 49–51). The results of the present study suggest that residues 190 and 230 play important roles in the antigenicity of EA H1N1 SIV and could be involved in the specificity of host cell recognition. Epitope identification demonstrated that an avian receptor residue at position 190 was essential for antigen recognition. In the present study, the single-amino-acid substitution N190D was found to influence the receptor-binding affinity for the human-type receptor of EA H1N1 SIV, suggesting that the regulation of receptor-binding affinity may be an important mechanism underlying the generation of immune escape variants of EA H1N1 SIV under natural conditions.

Our study found that complete neutralization resistance to two murine mAbs was conferred by the double substitution of N190D and I230M in HA or the single substitution of M269R in HA of EA H1N1 SIV. The decreased affinity for the human-type sialic acid receptor correlated with the antigenicity changes associated with the N190D substitution in the HA of the escape mutant. Given the possibility for antigen drift caused by the widespread presence of EA H1N1 SIV in pigs and the continuing sporadic introductions to humans, intensive monitoring of the effects of mutations in HA is necessary to predict variants with pandemic potential.

## MATERIALS AND METHODS

### Ethics statement and facility.

The study protocol was approved by the Committee on the Ethics of Animal Experiments of the Harbin Veterinary Research Institute of the Chinese Academy of Agricultural Sciences (Harbin, China) and conducted in strict accordance with the guide for the care and use of laboratory animals of the Ministry of Science and Technology of China (55). All animals were housed in a biosafety level 2 facility.

### Cells, viruses, and antibodies.

Human embryonic kidney HEK293T cells were maintained in Dulbecco’s modified Eagle’s medium (DMEM) supplemented with 10% fetal bovine serum (FBS). MDCK cells were cultured in DMEM containing 5% FBS. hTERT-PTECs were cultured in DMEM/F-12 medium supplemented with 10% FBS and epithelial cell growth factors (Lonza, Basel, Switzerland), as described previously (30). A/swine/Henan/11/2005 (H1N1) (HeN11) (GenBank accession numbers HQ541672 to HQ541679) was isolated from pigs in China. Virus was propagated in MDCK cells and titrated by the Reed-Muench method to determine the 50% tissue culture infectious dose (TCID_50_) (52).

Two murine mAbs against HA, designated 2B6 and 4C7, were generated by using the purified EA H1N1 SIV HeN11 protein as the immunogen. Both mAbs possessed high virus-neutralizing activities with strong antiviral infection abilities (protective efficacies), as verified in BALB/c mice (28). Chicken antisera against the respective viruses, including the wild-type virus, the escape mutant, and the single-site-directed mutant, were raised by immunization with oil-emulsified inactivated vaccines. Eight-week-old specific-pathogen-free chickens were immunized with 0.2 mL of a whole-virus inactivated oil-emulsified vaccine. Three weeks after vaccination, sera were collected from the chickens, and titers were determined by using the HI assay. Pig antisera against the corresponding viruses were prepared by inoculating 6-week-old pigs that were seronegative for H1 and H3 subtype SIVs. Three weeks after immunization, sera were collected from the pigs and treated with receptor-destroying enzyme prior to use in the HI assay.

### Selection of *in vitro* escape mutants.

The mAb-resistant mutants were selected by propagating the HeN11 virus in the presence of increasing concentrations of mAbs. In brief, mAbs were serially diluted 2-fold and mixed with the HeN11 virus. The virus-mAb mixtures at various mAb dilutions were incubated for 1 h at 37°C and then transferred to MDCK cells. After incubation for 1 h at 37°C under 5% CO_2_, the medium was replaced with virus growth medium containing 1 μg/mL tosylsulfonyl phenylalanyl chloromethyl ketone (TPCK)-trypsin, and the plates were then incubated at 37°C until HA activities were detected. The culture supernatants that were positive for virus and those with the highest viable concentrations of mAbs were then collected for the next passage. The mAb concentration was increased after each passage, and the initial selection process ended when the passaged virus was no longer neutralized by the respective mAbs. The virus was also propagated in the absence of mAbs in parallel to detect any passage-related mutations.

### Viral whole-genome sequencing.

The whole genome of the virus at each passage was sequenced by using an Applied Biosystems (Carlsbad, CA, USA) 3500xL genetic analyzer, as described previously (13). Briefly, viral RNA (vRNA) was extracted using a QIAamp viral RNA minikit (Qiagen, Germantown, MD, USA), and cDNA was synthesized from vRNA, reverse transcribed by using Uni12 primers, and amplified by PCR by using primers specific for each fragment (the primer sequences are available upon request).

### Homology modeling.

The SWISS-MODEL online server was used to construct a homology model of HA. Briefly, the amino acid sequence of HA was submitted to the server, and 50 templates were retrieved from the Protein Data Bank (PDB). The base-matched template (PDB accession number 6ONA) showed 76.47% sequence identity to the target HA. Three-dimensional models of the HA trimer were constructed, and the quality of the models was evaluated with Ramachandran plots. The model was visualized and analyzed by using the University of California, San Francisco, Chimera program (https://www.cgl.ucsf.edu/chimera/).

### Virus rescue.

A bidirectional eight-plasmid-based reverse genetic system for HeN11 was constructed to insert the corresponding gene into the vector pHW2000 (kindly provided by Richard Webby of St. Jude Children’s Research Hospital, Memphis, TN, USA) and used to rescue the virus as described previously (13). Reassortant viruses were rescued by transfecting cocultured human embryonic kidney HEK293T and MDCK cells with the recombinant plasmids, propagated in MDCK cells or 10-day-old specific-pathogen-free embryonated chicken eggs, followed by whole-genome sequencing to ensure the absence of unwanted mutations. The primer sequences for virus rescue are available upon request.

### Serological testing.

HI and neutralization reactivities were tested as previously described (53), and titers were determined as the highest dilution that completely inhibited viral hemagglutination activities.

### Receptor-binding assay.

The receptor preference of each tested virus was examined by using a solid-phase binding assay as described previously (54), using two different glycopolymers: an α-2,3-sialylglycopolymer (Neu5Acα2-3Galβ1-4GlcNAcβ1-pAP [*para*-aminophenyl]-α-PGA [α-polyglutamic acid]) (avian-type receptor) and an α-2,6-sialylglycopolymer (Neu5Acα2-6Galβ1-4GlcNAcβ1-pAP-α-PGA) (human-type receptor). Chicken antisera against the respective viruses were used as the primary antibodies, and a horseradish peroxidase-conjugated goat anti-chicken antibody (Sigma-Aldrich, St. Louis, MO, USA) was used as the secondary antibody. The absorbance was measured at a wavelength of 490 nm. Each experiment included three replicates.

### Viral growth kinetics.

MDCK cells and hTERT-PTECs were infected with the test viruses at multiplicities of infection of 0.001 and 0.01 in the presence of 1 μg/mL and 0.1 μg/mL TPCK-trypsin, respectively, at 37°C. Supernatants were collected at 12, 24, 36, 48, 60, and 72 hpi and stored at −80°C. The viral titers were determined by endpoint titration and expressed as the mean log_10_ TCID_50_ per milliliter ± the standard deviation (SD) from three independent experiments.

### Statistical analysis.

Data were analyzed by using two-way analysis of variance followed by Tukey’s multiple-comparison test in GraphPad Prism software (version 8.0; GraphPad Software, Inc., San Diego, CA, USA). A *P* value of <0.05 was considered statistically significant.

### Data availability.

The HA genes of the escape mutants were sequenced and have been deposited in GenBank under the accession numbers ON479265 and ON479266.
